# HLA-DQA1*05 Associates with Extensive Ulcerative Colitis at Diagnosis: An Observational Study in Children

**DOI:** 10.3390/genes12121934

**Published:** 2021-11-30

**Authors:** Jan Krzysztof Nowak, Aleksandra Glapa-Nowak, Aleksandra Banaszkiewicz, Barbara Iwańczak, Jarosław Kwiecień, Anna Szaflarska-Popławska, Urszula Grzybowska-Chlebowczyk, Marcin Osiecki, Jarosław Kierkuś, Magdalena Hołubiec, Justyna Chanaj-Kaczmarek, Andrzej Radzikowski, Jarosław Walkowiak

**Affiliations:** 1 Department of Pediatric Gastroenterology and Metabolic Diseases, Poznań University of Medical Sciences, Szpitalna 27/33, 60-572 Poznań, Poland; jan.nowak@ump.edu.pl (J.K.N.); glapa@ump.edu.pl (A.G.-N.); magdalena.holubiec@ump.edu.pl (M.H.); 2 Department of Pediatric Gastroenterology and Nutrition, Medical University of Warsaw, 02-091 Warszawa, Poland; aleksandra.banaszkiewicz@wum.edu.pl (A.B.); andrzej.radzikowski@uckwum.pl (A.R.); 3 Department and Clinic of Pediatrics, Gastroenterology and Nutrition, Wroclaw Medical University, 50-367 Wrocław, Poland; barbara.iwanczak@umed.wroc.pl; 4 Department of Pediatrics, Faculty of Medical Sciences in Zabrze, Medical University of Silesia in Katowice, 41-800 Zabrze, Poland; jkwiecien@sum.edu.pl; 5 Department of Pediatric Endoscopy and Gastrointestinal Function Testing, Collegium Medicum in Bydgoszcz, Nicolaus Copernicus University in Toruń, 85-067 Bydgoszcz, Poland; aszaflarska@wp.pl; 6 Department of Pediatrics, Faculty of Medical Sciences, Medical University of Silesia in Katowice, 40-055 Katowice, Poland; uchlebowczyk@sum.edu.pl; 7 The Department of Gastroenterology, Hepatology, Feeding Disorders and Paediatrics, The Children’s Memorial Health Institute, 04-730 Warsaw, Poland; M.Osiecki@IPCZD.Pl (M.O.); j.kierkus@med-net.pl (J.K.); 8 Department of Pharmacognosy, Poznan University of Medical Sciences, 4 Święcickiego Street, 60-781 Poznan, Poland; justyna.chanaj-kaczmarek@ump.edu.pl

**Keywords:** inflammatory bowel disease, colitis, Crohn’s disease, anti-drug antibodies, personalized medicine, high-resolution melting, major histocompatibility complex

## Abstract

The human leukocyte antigen (HLA) allele group HLA-DQA1*05 predisposes to ulcerative colitis (UC) and is associated with the development of antibodies against infliximab in patients with inflammatory bowel disease (IBD). Therefore, we hypothesized that the presence of HLA-DQA1*05 correlates with characteristics of pediatric IBD. Within a multi-center cohort in Poland, the phenotype at diagnosis and worst flare was established and HLA-DQA1*05 status was assessed enabling genotype-phenotype analyses. HLA-DQA1*05 was present in 221 (55.1%) out of 401 children with IBD (UC *n* = 188, Crohn’s disease *n* = 213). In UC, the presence of HLA-DQA1*05 was moderately associated with a large extent of colonic inflammation at diagnosis (E4 55% more frequent in HLA-DQA1*05-positive patients, *p* = 0.012). PUCAI at diagnosis (*p* = 0.078) and the time from UC diagnosis to the first administration of biologic treatment (*p* = 0.054) did not differ depending on HLA-DQA1*05 status. The number of days of hospitalization for exacerbation was analyzed in 98 patients for whom sufficient follow-up was available and did not differ depending on HLA-DQA1*05 carriership (*p* = 0.066). HLA-DQA1*05 carriers with CD were less likely to present with both stenosing and penetrating disease (B2B3, *p* = 0.048) and to have active disease proximal to the ligament of Treitz (L4a) at the worst flare (*p* = 0.046). Future research focusing on explaining and preventing anti-TNF immunogenicity should take into account that ADA may develop not only as an isolated reaction to anti-TNF exposure but also as a consequence of intrinsic differences in the early course of UC.

## 1. Introduction

Inflammatory bowel diseases (IBD) are life-long conditions affecting up to 0.5–1% of populations in developed countries. Patients typically experience diarrhea and abdominal pain, often requiring treatment with anti-tumor necrosis factor (anti-TNF) biologics. Infliximab has become a key IBD medication globally, with overall spending reaching billions of dollars. However, most patients develop anti-infliximab antibodies that may lead to treatment inefficacy.

The PANTS consortium recently showed that the allele group HLA-DQA1*05 associates with the development of antibodies against infliximab in patients with inflammatory bowel diseases (IBD) [1]. Since HLA-DQA1*05 is carried by 20–40% of Europeans, it is an actionable target providing an accessible foundation for personalized medicine in IBD. Compared with other nascent biomarkers derived from omics research [2], HLA-DQA1*05 assessment is potentially much simpler and cheaper. No randomized trials have yet been conducted but it seems that the presence of HLA-DQA1*05 could prompt more aggressive treatment, e.g., with a combination of infliximab and an immunosuppressant, or a different biologic.

Although the link between HLA-DQA1*05 and anti-drug antibody (ADA) development is statistically significant (rs2097432; HR 1.90, *p* = 5.88 × 10^−13^), the mechanisms mediating this effect remain uncertain [3]. Moreover, HLA-DQA1*05 is representative of a group of allelic variants, which is associated with the development of antibodies against infliximab or adalimumab, therefore four-digit HLA analysis could allow for even more detailed predictions [4,5]. 

Since HLA-DQA1*05 relates to celiac disease (and Grave’s disease), it can be argued that some of the pathophysiological mechanisms are shared between celiac autoimmunity and the generation of ADA [6]. Moreover, celiac disease (or Grave’s disease) is much less common in IBD patients than ADA development, so it can also be speculated that HLA-DQA1*05 facilitates the formation of ADA in a process dependent on IBD. The link between HLA-DQA1*05 and IBD characteristics can be explored in a setting free from potential confounding by anti-TNF agents, that is, at diagnosis, thus in childhood and adolescence. It was hypothesized that the presence of HLA-DQA1*05 correlates with greater severity of ulcerative colitis (UC) and Crohn’s disease (CD) at diagnosis, more rapid onset, and earlier need for immunosuppressive or biological treatment.

## 2. Materials and Methods

### 2.1. Patients and Setting

Patients for this cross-sectional study were recruited between 2016–2019 from seven Polish pediatric gastroenterology centers: Poznan University of Medical Sciences (Poznań, Poland), The Children’s Memorial Health Institute (Warsaw, Poland), Medical University of Warsaw (Warsaw, Poland), Wroclaw Medical University (Wrocław, Poland), Medical University of Silesia (Zabrze and Katowice, Poland), and Nicolaus Copernicus University (Bydgoszcz, Poland). The diagnosis of IBD was established using standard diagnostic criteria [7,8]. The inclusion criteria involved diagnosis of CD or UC as well as age ≥ 3 years and ≤18 years. Patients in life-threatening, severe general condition were excluded from the study. All the patients’ parents or guardians provided informed written consent and the study was approved by the Bioethical Committee of Poznan University of Medical Sciences (960/15 with the associated amendments) and adheres to the revised Declaration of Helsinki.

### 2.2. Disease Severity Evaluation

Disease activity was assessed using the Pediatric Ulcerative Colitis Activity Index (PUCAI) or Pediatric Crohn’s Disease Activity Index (PCDAI) at diagnosis and worst flare [9]. The worst flare was defined with the highest PUCAI or PCDAI result in the medical history. Serum albumin and C-reactive protein (CRP) concentrations at diagnosis and worst flare were obtained from medical records. Data were collected regarding systemic steroid intake with the total number of courses, immunosuppressive treatment with the age at first intake, biological therapy with the time at first infusion, and operative treatment with the age at first surgery. Localization and behavior of the disease were defined by the Paris Classification at diagnosis and worst flare [10] but these data were missing for some hospitalizations. Based on medical records, the total number of hospitalizations triggered by exacerbation was counted, with exacerbation defined by PUCAI ≥ 65 or PCDAI ≥ 50. The number of days spent in hospital for exacerbations was summarized for patients with a disease duration of at least one year. These exacerbation-related metrics were then divided by the number of years since diagnosis to obtain an individual estimate of severity. 

### 2.3. Genotyping

DNA was isolated from whole blood using Blood Mini (A&A Biotechnology, Gdynia, Poland). The primers proposed by Profaizer et al. [11] were used: forward 5′CAC GTC GCC TCT TAT GGT GTA; reverse 5′GAC TGT TCA AGT TAT GTT TTA GG. Reactions were prepared as follows: 1 μL of each specific primer (10 mM), 1 μL of water, 5 μL of FastStart Universal SYBR Green Master Mix (Sigma Aldrich, St. Louis, MO, USA) and 2 μL of genomic DNA for a total of 10 μL per reaction. The genotyping was performed using the Bio-Rad CFX96 thermocycler (Hercules, CA, USA) and the following thermo-profile: 95 °C for 5 min, 35 cycles of 10 s at 95 °C, followed by 30 s at 50 °C, 10 s at 72 °C with signal acquisition. The melting profile started with 5 min at 72 °C, then the temperature was increased from 65–95 °C for PCR product verification. Melting peaks representing the characteristic Tm of each particular DNA product were analyzed by CFX96 BioRad software. Inconclusive melting profiles, along with index positive and negative results, were sequenced (Sanger) to resolve uncertainties and serve as controls, respectively. As Sazonovs et al. demonstrated the relevance of HLA-DQA1*05 for ADA development in the dominant model, this work focused on the presence of HLA-DQA1*05, regardless of hetero- or homozygosity [1].

### 2.4. Statistical Analysis

The data were analyzed using Statistica 13.3 (TIBCO, Palo Alto, CA, USA) and R (R Software Foundation, Vienna, Austria). Z-scores for anthropometric parameters were calculated using reference values for the local population. The data are presented as mean ± mean or percentage, unless specified otherwise. The Mann-Whitney U test was used to compare continuous variables. The Fisher’s exact test was used for the comparison of binary parameters. A forward stepwise regression was built to compensate for confounding. The significance threshold was set at 0.05. 

## 3. Results

### 3.1. Cohort Characteristics

HLA-DQA1*05 status was assessed in 401 children with IBD, including 188 with UC and 213 with CD (Table 1). The typical patient with IBD was diagnosed at 7–16 years and followed up for 3 years on average. Children with UC were younger and had better nutritional status at diagnosis compared to CD, as well as exhibiting lower CRP levels both at diagnosis and worst flare (Table 1). Most IBD patients received steroids and immunosuppressants. However, UC was more frequently treated with systemic steroids while azathioprine was used more often in CD. A greater fraction of CD patients received biological therapy or needed to undergo IBD-related surgery. Most children (55.1%) carried the HLA-DQA1*05 allele group.

### 3.2. HLA-DQA1*05 Associates with Extensive Colitis at Diagnosis

In UC, the presence of HLA-DQA1*05 was associated with pancolitis at diagnosis (E4, which was 55% more frequent in HLA-DQA1*05-positive patients, *p* = 0.012; Table 2). However, pancolitis was also associated with the male gender (*p* = 0.038). In forward stepwise regression analysis of data from UC patients, HLA-DQA1*05 but not gender was weakly associated with Paris E4 status at diagnosis (R2 = 0.03, *p*_model_ = 0.0077; β = 0.1939, 95% CI 0.0520–0.3358). There was no other evidence of a more severe UC course at diagnosis in children positive for HLA-DQA1*05. The time from UC diagnosis to first administration of immunosuppressive (*p* = 0.133) or biologic treatment (*p* = 0.054) did not differ depending on HLA-DQA1*05 status. HLA-DQA1*05 patients also did not have lower mass, height (*p* = 0.060) or BMI at diagnosis, PUCAI at diagnosis (*p* = 0.078) or worst flare and had CRP levels similar to HLA-DQA1*05-negative peers both at diagnosis and worst flare. The number of days of hospitalization for exacerbation was analyzed in 98 patients with UC for whom sufficient follow-up was available and did not differ depending on HLA-DQA1*05 status (*p* = 0.066). Four patients with UC received immunosuppressants prior to diagnosis. All were HLA-DQA1*05 positive and half of them developed pancolitis at diagnosis.

### 3.3. HLA-DQA1*05 Might Relate to the CD Phenotype in Children

HLA-DQA1*05 carriers with CD were less likely to present with both stenosing and penetrating disease (B2B3, *p* = 0.048) and have active disease proximal to the ligament of Treitz (L4a) at worst flare (*p* = 0.046; Table 3). Nominally more frequent perianal involvement was not significantly associated with HLA-DQA1*05 (13.4% vs. 5.0%, *p* = 0.054) or age at first CD surgery (13.2 ± 2.2 years vs. 14.6 ± 3.3 years, *p* = 0.051). No other relationships were found between HLA-DQA1*05 and the clinical characteristics of CD. 

## 4. Discussion

This study explored the clinical correlation of carrying the HLA-DQA1*05 allele group in 401 children and adolescents with IBD focusing on disease severity, age at onset, and the time to initiation of immunosuppressive or biologic treatment. HLA-DQA1*05 positively associated with the broad extent of colonic inflammation at diagnosis of UC. Therefore, HLA-DQA1*05 seems relevant for UC beyond ADA generation. It may be speculated that more extensive colitis at diagnosis, which is associated with HLA-DQA1*05, predisposes to ADA development.

### 4.1. Gender and the Relationship between HLA-DQA1*05 and Colitis Extent

The observation that males were over 40% more frequently HLA-DQA1*05-positive than females leads to the question of whether the main finding can be confounded by gender. As indicated by regression analysis, the link between HLA-DQA1*05 and the extent of colitis proved stronger than with gender. Moreover, the male gender is a known factor for a severe course of UC, therefore the larger percentage of men in the E4 group is unsurprising [12]. However, the interaction between gender and HLA-DQA1*05 was not excluded in this study, so one could speculate that there is an interaction between gender and HLA-DQA1*05 in IBD development.

### 4.2. Celiac Disease and the Relationship between HLA-DQA1*05 and the Extent of Colitis

Five patients with UC had celiac disease. Of these five patients four carried HLA-DQA1*05. Pancolitis (E4) was found in only one of these children suggesting that the relationship between HLA-DQA1*05 and more severe colitis at diagnosis is not dependent on factors directly predisposing to celiac disease. However, any link between celiac disease and the occurrence of ADA would need to be explored separately.

### 4.3. HLA-DQA1*05 Effect in UC, but Not CD

Sazonovs et al. initially reported the link between HLA-DQA1*05 in CD, later confirming the association in both CD and UC within a validation sub-study, revealing similar hazard ratios for both diseases (HR = 2.26 in CD; HR = 2.02 in UC) [5]. However, our study identified a relationship between disease severity and HLA-DQA1*05 only in UC, despite a greater number of patients with CD (over 200). This calls for hypotheses explaining the impact of HLA-DQA1*05 on UC but not CD severity at diagnosis. 

Molecular dissection of UC vs. CD is challenging because of the many similarities that these entities share in omics-based research. Genome-wide association studies found that the single nucleotide polymorphism rs6927022 located immediately downstream from HLA-DQA1 (1 kbp) strongly correlates with the development of UC but not CD [13]. In some analyses, rs6927022 was considered to be the genetic loci most strongly associated with UC [14].

It is also interesting that HLA-DQA1*05:01 remains in linkage disequilibrium with HLA-DRB1*03:01 (D’ = 0.75 according to LDpair; European populations), which is associated with an increased risk of Crohn’s disease in a study focusing on four-digit HLA typing by microarrays in over 30,000 IBD patients [15]. Yet, whereas HLA-DQA1*05 in numerous cases should predispose to disease occurrence and ADA formation in both UC and CD, UC alone predisposes to more severe disease. 

Other UC-specific genetic predispositions include WNT2, a gene involved in epithelial proliferation and the modulation of inflammatory responses. Furthermore, some CD-specific genetic predispositions are related to bacterial antigen sensing (TLR4, NOD2), autophagy (ATG16L1), the killing of bacteria (NCF4), and the interferon response (IFNGR2). Despite the above relationships and several other differences, the HLA locus containing HLA-DQA1 and HLA-DQB1 seems to be key to the observed lack of a relationship with severity at onset in CD.

### 4.4. HLA-DQA1*05 Allele Group at Four-Digit Resolution

The latest data indicate an opportunity to develop and use higher-resolution assessment to obtain more precise measurements regarding the generation of ADA. Whereas HLA-DQA1*05:01, HLA-DQB1*02:01, and HLA-DRB1*03:01 remain in linkage disequilibrium and associate with infliximab immunogenicity, HLA-DQA1*05:05, HLA-DQB1*03:01, and HLA-DRB1*11:01 (also in linkage disequilibrium) associate mainly with adalimumab immunogenicity [4]. However, HLA-DQA1*05:05 alone could explain only a modest fraction of all cases of anti-adalimumab ADA development [5]. As highlighted by Powell-Doherty et al. [4], it may be worthwhile to investigate HLA-DRB1*03:01 (infliximab) and HLA-DRB1*11:01 (adalimumab) in the context of ADA generation. It should be considered, however, that the frequency of HLA-DRB1*03:01 is nearly four times lower than that of HLA-DQA1*05.

### 4.5. Strengths and Limitations of the Current Analysis

Apart from focusing on HLA-DQA1*05 without four-digit HLA allele genotyping, this study is limited by the lack of long-term data on the response to infliximab or ADA development, precluding validation of the PANTS study. Also, even though the dominant model has been established for the influence of HLA-DQA1*05 on ADA development, investigation of the additive model could also be of value. Nevertheless, the results presented in this work reveal an important relationship between HLA-DQA1*05 and UC extent at diagnosis. This was accomplished in a large cohort with rich phenotype data, providing a new dimension to our understanding of the relationship between HLA-DQA1*05 and anti-TNF immunogenicity. 

### 4.6. The Cohort Captures a Transition Period in Infliximab Use in Polish Children

This study describes the clinical characteristics and treatment modalities of a large pediatric IBD cohort from Poland, covering a transition period in the availability of biologics for pediatric IBD. Initially, the access to therapeutic programs was rather limited, requiring high PCDAI scores for eligibility but the introduction of biosimilars gradually increased the availability of anti-TNF agents for this cohort. Regardless of the improved availability, the patients within the cohort were still treated using a step-up strategy, and this transition is unlikely to confound analyses of time from diagnosis to treatment with anti-TNF agents.

Another noteworthy aspect of the cohort is that patients with CD had poorer nutritional status and stronger inflammation at diagnosis compared to UC and that almost 80% were exposed to immunosuppressants relatively early. 

### 4.7. New Research Directions in UC

The relationship between HLA-DQA1*05 and greater PUCAI (mean 46 vs. 40, *p* = 0.078) as well as lower height Z-score at UC diagnosis (mean −0.06 vs. 0.18, *p* = 0.060) would require additional investigation in even larger cohorts. Similarly, such research could be helpful to determine links between HLA-DQA1*05 and the shorter time from diagnosis to first biologic (mean 16.3 vs. 27.6 months, *p* = 0.054) and the greater number of days of hospitalization for exacerbation per year in UC (mean 9.22 vs. 5.45 days, *p* = 0.066). Our study shows that if such effects exist, they are likely subtle. As already discussed, there were no similar results in CD. Future studies could focus on targeting links between HLA genetics and CD location at diagnosis and worst flare, with and without the use of anti-TNF agents, to potentially uncover disease subtypes. Analysis of HLA-DQA1*05 allele homo- and heterozygosity in the context of polygenic risk scores could help elucidate the interactions between gender and HLA-DQA1*05-related pancolitis in UC.

## 5. Conclusions

In summary, HLA-DQA1*05 correlates with a greater extent of colonic inflammation at diagnosis in children with UC. Future research focusing on explaining and preventing anti-TNF immunogenicity should take into account that ADA may develop not only as an isolated reaction to anti-TNF exposure but as a consequence of intrinsic differences in the early course of UC.

## Figures and Tables

**Table 1:** Group characteristics.

	Ulcerative Colitis	Crohn’s Disease	*p*
*n*	188	213	
Age at inclusion, years	13.9 ± 3.5	14.8 ± 2.6	0.046
Age at diagnosis, years	11.3 ± 4.4	12.0 ± 3.7	0.383
Age at worst flare, years	12.7 ± 4.0	13.4 ± 3.1	0.276
Duration of disease, years	2.7 ± 2.9	3.5 ± 8.4	0.214
Sex	46.3% female	39.9% female	0.225
Mass at diagnosis, kg	41.1 ± 17.9	39.8 ± 16.6	0.449
Mass at diagnosis, Z-score	−0.32 ± 1.12	−0.62 ± 1.24	0.004
Height at diagnosis, kg	148 ± 26	149 ± 22	0.657
Height at diagnosis, Z-score	0.04 ± 1.14	−0.36 ± 1.33	0.001
BMI at diagnosis, kg/m^2^	17.7 ± 3.0	17.2 ± 3.6	0.018
BMI at diagnosis, Z-score	−0.40 ± 0.99	−0.61 ± 1.20	0.007
Score at diagnosis	PUCAI 43 ± 19	PCDAI 34 ± 16	
Score at worst flare	PUCAI 50 ± 19	PCDAI 40 ± 15	
CRP at diagnosis, mg/L	10.5 ± 22.5	21.9 ± 30.4	4.72 × 10^−9^
CRP at worst flare, mg/L	14.9 ± 34.6	27.4 ± 42.7	2.13 × 10^−7^
Steroids used prior to inclusion	71.3%	54.0%	4.4 × 10^−4^
Immunosuppressants used before inclusion	58.8%	78.9%	2 × 10^−5^
Biologics used prior to inclusion	25.5%	49.8%	<1 × 10^−5^
Surgery during follow-up	2.1%	13.1%	3 × 10^−5^
HLA-DQA1*05 positive	58.0%	52.6%	0.315

**Table 2:** Clinical characteristics of ulcerative colitis in children depending on HLA-DQA1*05 status.

	HLA-DQA1*05 Negative	HLA-DQA1*05 Positive	*p*
*n*	110	78	
Age at inclusion	13.8 ± 3.3	14.0 ± 3.6	0.393
Age at diagnosis	10.8 ± 4.4	11.7 ± 4.4	0.166
Duration of disease	2.9 ± 2.7	2.6 ± 3.0	0.164
Gender	55.7% female	39.4% female	0.038
Mass at diagnosis, Z-score	−0.17 ± 1.17	−0.42 ± 1.07	0.115
Height at diagnosis, Z-score	0.18 ± 1.16	−0.06 ± 1.12	0.060
BMI at diagnosis, Z-score	−0.32 ± 1.06	−0.47 ± 0.94	0.346
Mass at worst flare, Z-score	−0.25 ± 1.18	−0.29 ± 1.15	0.736
Height at worst flare, Z-score	0.06 ± 1.04	0.01 ± 1.06	0.485
BMI at worst flare, Z-score	−0.44 ± 1.51	−0.35 ± 1.14	0.894
PUCAI at diagnosis	40 ± 20	46 ± 18	0.078
PUCAI at worst flare	48 ± 20	51 ± 18	0.499
CRP at diagnosis	9.2 ± 19.4	11.4 ± 24.6	0.797
CRP at worst flare	12.7 ± 25.3	16.5 ± 40.2	0.457
E1 at diagnosis	12.7%	7.3%	0.315
E2 at diagnosis	19.0%	16.5%	0.700
E3 at diagnosis	17.7%	13.8%	0.541
E4 at diagnosis	35.4%	55.0%	0.012
S0 at diagnosis	59.5%	62.4%	0.762
S1 at diagnosis	19.0%	21.1%	0.854
E1 at worst flare	6.3%	2.8%	0.284
E2 at worst flare	13.9%	14.7%	1.000
E3 at worst flare	17.7%	8.3%	0.070
E4 at worst flare	35.4%	48.6%	0.076
S0 at worst flare	45.6%	48.6%	0.767
S1 at worst flare	26.6%	24.8%	0.866
Steroids	69.6%	72.5%	0.745
Immunosuppressants	59.5%	58.3%	0.882
Time from diagnosis to first immunosuppressant, mo.	9.7 ± 19.9	9.2 ± 18.9	0.133
Age at first immunosuppressant	10.5 ± 4.0	11.3 ± 4.6	0.199
Biologics	22.8%	27.5%	0.501
Time from diagnosis to first biologic, mo.	27.6 ± 25.3	16.3 ± 14.3	0.054
Age at first biologic	11.7 ± 4.1	11.1 ± 4.7	0.702
Surgery	2.5%	1.8%	1.000
Time from diagnosis to first IBD surgery	23.7 ± 16.8	8.8 ± 11.2	0.386
Age at first IBD surgery	11.6 ± 4.4	11.2 ± 6.2	0.846
Number of hospitalizations for exacerbation/year, *n* = 98	0.84 ± 0.80	1.11 ± 1.09	0.318
Days of hospitalization for exacerbation/year, *n* = 98	5.45 ± 5.86	9.22 ± 9.34	0.066

**Table 3:** Clinical characteristics of Crohn’s disease in children depending on HLA-DQA1*05 status.

	HLA-DQA1*05 Negative	HLA-DQA1*05 Positive	*p*
*n*	101	112	
Age at inclusion	14.7 ± 2.8	14.9 ± 2.4	0.797
Age at diagnosis	11.7 ± 3.8	12.2 ± 3.6	0.384
Duration of disease	4.2 ± 11.9	2.7 ± 2.5	0.593
Gender	39.6% female	40.2% female	1.000
Mass at diagnosis, Z-score	−0.70 ± 1.22	−0.54 ± 1.26	0.398
Height at diagnosis, Z-score	−0.41 ± 1.48	−0.32 ± 1.18	0.510
BMI at diagnosis, Z-score	−0.69 ± 1.07	−0.53 ± 1.30	0.578
Mass at worst flare, Z-score	−0.80 ± 1.24	−0.64 ± 1.22	0.324
Height at worst flare, Z-score	−0.50 ± 1.36	−0.48 ± 1.19	0.849
BMI at worst flare, Z-score	−0.75 ± 1.17	−0.56 ± 1.23	0.271
PCDAI at diagnosis	34 ± 18	34 ± 15	0.927
PCDAI at worst flare	38 ± 15	42 ± 15	0.157
CRP at diagnosis	20.4 ± 26.0	23.2 ± 34.0	0.933
CRP at worst flare	20.4 ± 25.9	33.9 ± 53.1	0.215
L1 at diagnosis	24.0%	24.1%	1.000
L2 at diagnosis	17.0%	19.6%	0.723
L3 at diagnosis	48.0%	44.6%	0.680
L4a at diagnosis	14.0%	7.1%	0.118
L4b at diagnosis	3.0%	4.5%	0.725
B1 at diagnosis	69.0%	70.5%	0.881
B2 at diagnosis	8.0%	5.4%	0.582
B3 at diagnosis	7.0%	10.7%	0.471
B2B3 at diagnosis	4.0%	0%	0.048
G0 at diagnosis	67.0%	71.4%	0.551
G1 at diagnosis	15.0%	15.2%	1.000
P at diagnosis	8.0%	9.8%	0.810
L1 at worst flare	17.0%	19.6%	0.723
L2 at worst flare	13.0%	12.5%	1.000
L3 at worst flare	45.0%	42.9%	0.783
L4a at worst flare	13.0%	4.5%	0.046
L4b at worst flare	6.0%	2.7%	0.312
B1 at worst flare	56.0%	53.6%	0.783
B2 at worst flare	7.0%	9.8%	0.623
B3 at worst flare	9.0%	10.7%	0.819
B2B3 at worst flare	4.0%	0.9%	0.191
G0 at worst flare	57.0%	59.8%	0.780
G1 at worst flare	16.0%	15.2%	1.000
P at worst flare	5.0%	13.4%	0.058
Steroids	52.5%	55.4%	0.682
Immunosuppressants	77.2%	80.4%	0.617
Time from diagnosis to first immunosuppressant	9.4 ± 17.3	7.0 ± 14.8	0.406
Age at first immunosuppressant	12.0 ± 3.9	12.3 ± 3.4	0.923
Biologics	45.5%	53.6%	0.273
Time from diagnosis to first biologic	23.8 ± 26.5	20.1 ± 23.0	0.623
Age at first biologic	12.9 ± 3.4	13.5 ± 2.7	0.626
Surgery	13.9%	12.5%	0.840
Time from diagnosis to first IBD surgery	27.5 ± 31.4	24.4 ± 23.5	1.000
Age at first IBD surgery	14.6 ± 3.3	13.2 ± 2.2	0.051
Number of hospitalizations for exacerbation/years, *n* = 132	0.69 ± 0.63	0.60 ± 0.67	0.317
Days of hospitalization for exacerbation/year, *n* = 132	6.3 ± 7.7	5.5 ± 6.1	0.622

## Data Availability

The data will be available from the corresponding author at reasonable request.
